# Association of Pharmaceutical Industry Marketing of Opioid Products With Mortality From Opioid-Related Overdoses

**DOI:** 10.1001/jamanetworkopen.2018.6007

**Published:** 2019-01-18

**Authors:** Scott E. Hadland, Ariadne Rivera-Aguirre, Brandon D. L. Marshall, Magdalena Cerdá

**Affiliations:** 1 Division of General Pediatrics, Department of Pediatrics, Boston University School of Medicine, Boston, Massachusetts; 2 Department of Pediatrics, Boston Medical Center, Boston, Massachusetts; 3 Grayken Center for Addiction, Boston Medical Center, Boston, Massachusetts; 4 Department of Emergency Medicine, School of Medicine, University of California at Davis, Sacramento; 5 Department of Population Health, New York University School of Medicine, New York; 6 Department of Epidemiology, Brown University School of Public Health, Providence, Rhode Island

## Abstract

**Importance:**

Prescription opioids are involved in 40% of all deaths from opioid overdose in the United States and are commonly the first opioids encountered by individuals with opioid use disorder. It is unclear whether the pharmaceutical industry marketing of opioids to physicians is associated with mortality from overdoses.

**Objective:**

To identify the association between direct-to-physician marketing of opioid products by pharmaceutical companies and mortality from prescription opioid overdoses across US counties.

**Design, Setting, and Participants:**

This population-based, county-level analysis of industry marketing information used data from the Centers for Medicare & Medicaid Services Open Payments database linked with data from the Centers for Disease Control and Prevention on opioid prescribing and mortality from overdoses. All US counties were included, with data on overdoses from August 1, 2014, to December 31, 2016, linked to marketing data from August 1, 2013, to December 31, 2015, using a 1-year lag. Statistical analyses were conducted between February 1 and June 1, 2018.

**Main Outcomes and Measures:**

County-level mortality from prescription opioid overdoses, total cost of marketing of opioid products to physicians, number of marketing interactions, opioid prescribing rates, and sociodemographic factors.

**Results:**

Between August 1, 2013, and December 31, 2015, there were 434 754 payments totaling $39.7 million in nonresearch-based opioid marketing distributed to 67 507 physicians across 2208 US counties. After adjustment for county-level sociodemographic factors, mortality from opioid overdoses increased with each 1-SD increase in marketing value in dollars per capita (adjusted relative risk, 1.09; 95% CI, 1.05-1.12), number of payments to physicians per capita (adjusted relative risk, 1.18; 95% CI, 1.14-1.21, and number of physicians receiving marketing per capita (adjusted relative risk, 1.12; 95% CI, 1.08-1.16). Opioid prescribing rates also increased with marketing and partially mediated the association between marketing and mortality.

**Conclusions and Relevance:**

In this study, across US counties, marketing of opioid products to physicians was associated with increased opioid prescribing and, subsequently, with elevated mortality from overdoses. Amid a national opioid overdose crisis, reexamining the influence of the pharmaceutical industry may be warranted.

## Key Points

**Question:**

To what extent is pharmaceutical industry marketing of opioids to physicians associated with subsequent mortality from prescription opioid overdoses?

**Findings:**

In this population-based, cross-sectional study, $39.7 million in opioid marketing was targeted to 67 507 physicians across 2208 US counties between August 1, 2013, and December 31, 2015. Increased county-level opioid marketing was associated with elevated overdose mortality 1 year later, an association mediated by opioid prescribing rates; per capita, the number of marketing interactions with physicians demonstrated a stronger association with mortality than the dollar value of marketing.

**Meaning:**

The potential role of pharmaceutical industry marketing in contributing to opioid prescribing and mortality from overdoses merits ongoing examination.

## Teaser

This population-based, cross-sectional study examines whether there is an association between direct-to-physician marketing of opioid products by pharmaceutical companies and mortality from prescription opioid overdoses across US counties.

## Introduction

Mortality from opioid-related overdoses continues to increase in the United States.^1^ Although heroin and illicitly manufactured fentanyl citrate have contributed to an increasing number of overdoses in many settings, prescription opioids are involved in 40% of all opioid overdoses^2^ and are frequently the first opioids that individuals encounter before subsequently transitioning to the use of illicit opioids.^3^ Physician prescribers are the most frequent source of prescription opioids for individuals who use opioids nonmedically.^4^ Despite an overall reduction in opioid prescribing in the United States since 2010, current prescribing rates remain 3 times higher than in 1999 and have not decreased as rapidly in regions experiencing high levels of mortality from opioid-related overdoses.^5,6^ Therefore, a central strategy of the national response to the overdose crisis has been to reduce the number of opioids prescribed by physicians.^7^

The potential influence of the pharmaceutical industry on opioid prescribing and mortality from overdoses has received substantial recent attention in the lay media.^8^ Direct-to-physician marketing by pharmaceutical companies is widespread in the United States and is associated with increased prescribing of the marketed products.^9,10,11^ Between 2013 and 2015, approximately 1 in 12 US physicians received opioid-related marketing; this proportion was even higher for family physicians, among whom 1 in 5 received opioid-related marketing.^12^ A prior study found that, among physicians providing care to Medicare patients, marketing was associated with increased prescribing of opioids.^11^ However, it is unclear to what extent such marketing may be associated nationally with opioid prescribing and, consequently, with deaths from overdoses.

Prior research demonstrates that the introduction and marketing of opioid products may be associated with subsequent mortality from overdoses.^13,14,15^ However, to our knowledge, no prior studies have examined mortality from prescription opioid overdoses in association with total marketing of opioid products. Drawing on recent US county-level data, we sought to conduct preliminary analyses into the extent to which opioid marketing is associated with subsequent prescribing and, in turn, with opioid-related deaths from overdoses. We hypothesized that counties receiving more marketing subsequently experience a higher rate of deaths from prescription opioid overdoses and that this association is mediated by an increase in county opioid prescribing rates.

## Methods

The study was approved by the Brown University Institutional Review Board. Patient consent was waived as the data on overdoses were deidentified and the data on pharmaceutical company payments and county-level prescribing rates were publicly available. The study followed the Strengthening the Reporting of Observational Studies in Epidemiology (STROBE) reporting guideline.^16^

### Data Sources

We linked county-level information from August 1, 2013, to December 31, 2016, across 3 national databases. From the first data source, the Centers for Disease Control and Prevention Wide-Ranging Online Data for Epidemiological Research Restricted-Use Mortality Files, we extracted data on deaths from overdoses for all counties in all 50 states and the District of Columbia.^6^ Consistent with national definitions,^1^ we identified deaths from prescription opioid drug overdoses using the *International Statistical Classification of Diseases and Related Health Problems, Tenth Revision* underlying cause-of-death codes for unintentional overdose (codes X40-X44), suicide by drug self-poisoning (codes X60-X64), homicide by drug poisoning (code X85), and deaths of undetermined intent (codes Y10-Y14) involving prescription opioids (codes T40.2-T40.4).

From the second data source, the Centers for Medicare & Medicaid Services Open Payments database, we extracted every transfer of value (“payment”) in marketing from a pharmaceutical company to a physician as mandated by the recent Physician Payments Sunshine Act.^17^ Each entry in the Open Payments database includes the monetary value of the payment in dollars, the medication(s) being marketed, the type of marketing (ie, meals, travel costs, speaking fees, honoraria, consulting fees, or educational costs), and the physician practice location. Practice locations were geocoded to determine their county (Geolytics Inc). We extracted data for all payments that included a US Food and Drug Administration–approved opioid product listed by brand or generic name.^18^ We included only nonresearch marketing because research payments are often made to support clinical trials and are frequently provided to nonpracticing physician researchers.

From a third database, available from the Centers for Disease Control and Prevention National Center for Injury Prevention and Control, we extracted data on opioid prescribing rates (including initial prescriptions and refills) dispensed at retail pharmacies.^19^ Prescribing rates were available for 87% to 94% of counties across years; we used multiple imputation methods to generate prescribing rates for counties in which data were missing.

### Variables

The primary outcome was the county-level annual death counts from prescription opioid overdoses with a population offset. The primary exposure was county-level annual pharmaceutical company opioid marketing to physicians, which we defined in the following 3 ways: (1) marketing value in dollars per 1000 county population, (2) number of payments to physicians per 1000 county population, and (3) number of physicians receiving marketing per 1000 county population. To obtain annual mean values for opioid marketing, we extrapolated from the final 5 months of 2013 (because data were available only for August onward for 2013) and used full-year data for 2014 and 2015. We also examined the role of county-level annual opioid prescribing rates per 100 population as a potential mediator of the association between opioid marketing and mortality from prescription opioid overdoses.

Covariates included time-varying, county-level sociodemographic characteristics, including county composition according to sex (percentage of individuals in a county who were classified as male), age (mean in years), race/ethnicity (classified according to the race/ethnicity exceeding 50% of the county composition, with counties not exceeding this cutoff classified as “mixed” counties), unemployment (expressed as a percentage), median household income (expressed in thousands of US dollars), poverty (percentage of adult population living below the federal poverty limit), educational level (percentage of population aged ≥25 years without education beyond high school), and Gini index of income inequality (ranging from 0, representing perfect income equality [ie, all incomes within a county are the same], to 1, representing perfect inequality [ie, 1 individual within a county holds all the county’s income, and all others in the same county have no income]) from the US Census Bureau American Community Survey,^20,21,22,23^ as well as metropolitan or nonmetropolitan location from the US Department of Agriculture 2013 Rural-Urban Continuum Codes classification scheme.^24^ Covariates were selected based on prior literature highlighting their association with deaths from overdoses; in particular, data demonstrate that mortality from overdoses is elevated in regions with greater economic distress (ie, higher unemployment, lower median household income, greater poverty, lower educational level, and greater income inequality).^25^

### Statistical Analysis

Statistical analyses were conducted between February 1 and June 1, 2018. We used negative binomial regression to measure longitudinal associations between opioid marketing and mortality rates from prescription opioid overdoses across counties. The negative binomial model was selected to address overdispersion among mortality rates; this approach provides effect sizes ranging from greater than 0 to infinity, with confidence intervals that do not overlap with unity representing a statistically significant finding.^26^ We introduced a 1-year lag between mortality rates and marketing to address the possibility of reverse causality by ensuring that marketing of opioids to physicians preceded observed mortality rates. We estimated separate models using each of the 3 measures of opioid marketing described as the exposure of interest. Multivariable analyses were adjusted for all the county-level sociodemographic covariates described. All models used robust SEs clustered at the county level, with the natural log-transformed population as the offset variable.

We conducted a mediation analysis to identify whether county-level opioid prescribing was an intermediate in the lagged cross-sectional association between marketing of opioids to physicians and mortality from overdoses.^27^ We first measured the association between each of the opioid marketing measures and prescribing rates using censored regression Tobit models. Tobit models, which estimate linear associations in which the dependent variable is left or right censored, were selected to address the large number of county prescribing rates with fewer than 15 prescriptions per 100 population.^28^ As in ordinary least-squares linear regression, Tobit models provide effect sizes ranging from negative infinity to positive infinity, and confidence intervals that do not overlap with 0 represent a statistically significant finding. A 1-year lag was introduced between prescribing rates and marketing. All models were adjusted for county-level sociodemographic covariates. Using adjusted effect sizes of the association between opioid marketing and mortality from prescription opioid overdoses obtained from the negative binomial regression models described, we used the *paramed* command in Stata, version 15.1 (StataCorp) to estimate the natural indirect (mediated) association of opioid prescribing and total (unmediated) direct association of opioid marketing with mortality from overdoses.^27^ We calculated percentage mediation as follows: % medication = [ln (natural indirect effect)/ln (total effect)] × 100.

Finally, we used multiple imputation to generate estimates for prescribing rates in counties in which data were missing (ranging from 6% to 13% of all counties across study years). Using predictive mean matching and specifying sequential chained equations, we created 20 data sets in which missing county prescribing rates were imputed. We then repeated the multivariable Tobit model linking opioid marketing to opioid prescribing rates across counties using the 20 complete data sets. The mediation analysis was also repeated for all 20 complete data sets. Results of analyses were pooled according to the Rubin rules.^29^

We conducted data management using Tableau Desktop, version 10.0 (Tableau Software) and analyses using Stata, version 15.1. We computed adjusted relative risk (aRR) ratios and 95% CIs by exponentiating parameter estimates. All 95% CIs were calculated using SEs clustered at the county level. All statistical tests were 2-sided and considered significant at *P* < .05.

## Results

In this population-based cross-sectional study, we found that, between August 1, 2013, and December 31, 2015, there were 434 754 payments totaling $39.7 million in nonresearch-based opioid marketing distributed to 67 507 physicians across 2208 US counties. The Figure displays the mortality rates from prescription opioid overdoses across US counties, the marketing value in dollars per 1000 county population, the number of payments to physicians per 1000 county population, and the number of physicians receiving opioid marketing per 1000 county population. The characteristics of the counties receiving marketing in comparison with those receiving no marketing are shown in eTable 1 in the Supplement.

**Figure.**
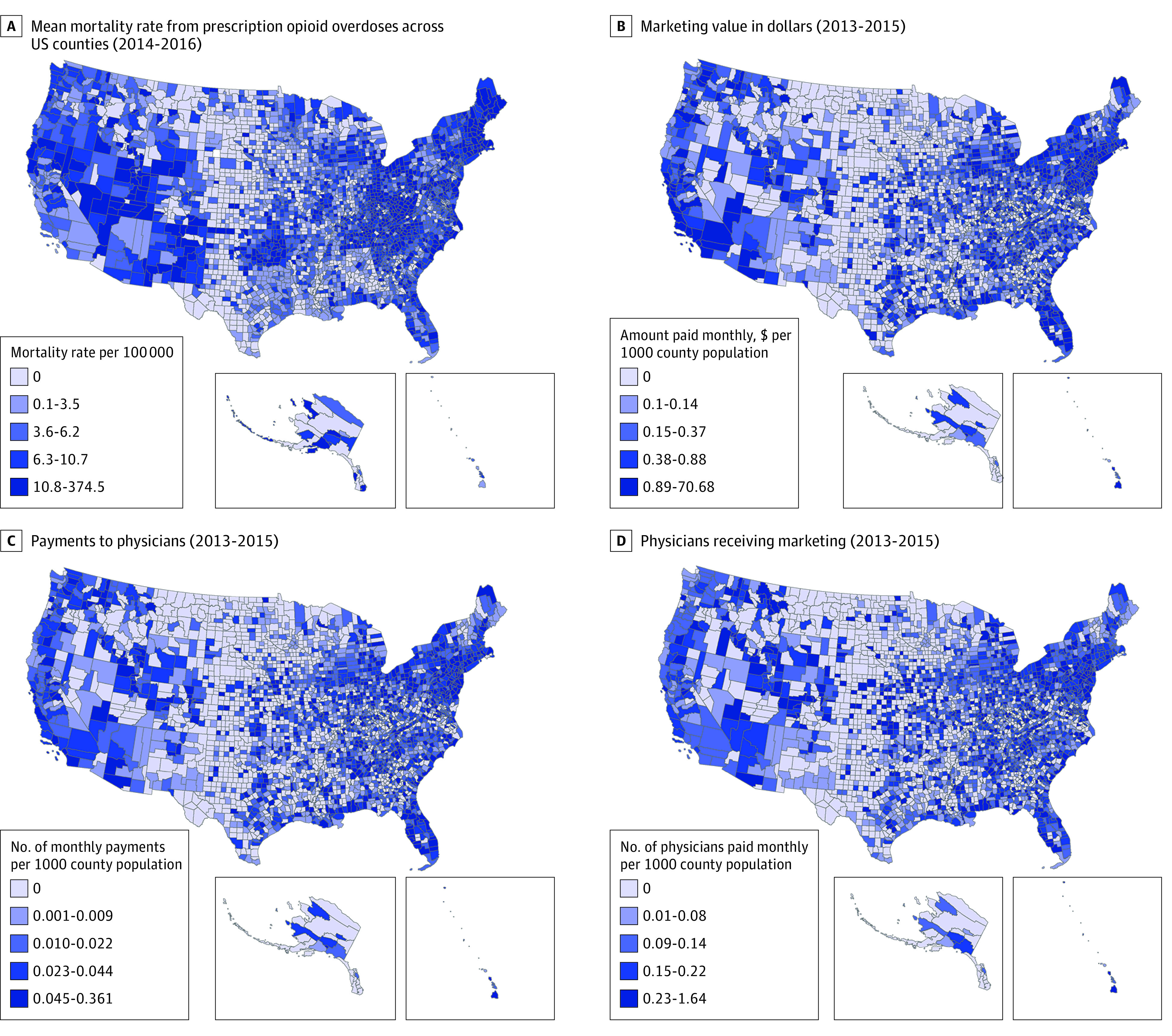
Mortality Rates From Prescription Opioid Overdoses in 2014-2016 and Marketing of Opioids by Pharmaceutical Companies to Physicians in 2013-2015

Table 1 displays the per capita prescription opioid marketing costs, the opioid prescribing rates, and the mortality rates from prescription opioid overdoses according to county-level covariates.^20,21,22,23^ Overall, the mean (SD) overdose mortality rate was 7.4 (9.0) per 100 000 person-years across counties. The mean (SD) marketing cost per county was valued at $1.57 ($5.29) per 1000 individuals and accounted for a mean (SD) of 0.03 (0.04) payments per 1000 individuals distributed to a mean (SD) of 0.01 (0.01) physicians per 1000 individuals. Opioid marketing dollars were most highly concentrated in counties with a lower percentage of individuals aged 65 years or younger and where the race/ethnicity composition was mixed; payments were also concentrated in counties marked by a higher prevalence of high school completion, greater unemployment, lower poverty, higher median household income, lower income inequality, and metropolitan location. The Northeast had the highest concentration of opioid marketing among US Census regions, and the Midwest had the lowest.

**Table 1.** Characteristics of Counties Receiving Opioid Marketing During 2013-2015 and Subsequent Opioid Prescribing and Mortality From Overdoses During 2014-2016^a^

Characteristic	Total Cost of Marketing, $ per 1000 Population, Mean (SD)	Total No. of Payments, per 1000 Population, Mean (SD)	No. of Physicians With ≥1 Payment, per 1000 Population, Mean (SD)	Opioid Prescribing Rate, per 100 Population, Mean (SD)	Overdose Mortality Rate, per 100 000 Person-Years, Mean (SD)
All counties receiving marketing	1.57 (5.29)	0.03 (0.04)	0.01 (0.01)	90.0 (42.8)	7.4 (9.0)
Age					
<15% aged >65 y (n = 1003)	2.36 (6.16)	0.04 (0.04)	0.01 (0.01)	85.8 (39.4)	7.4 (8.5)
≥15% aged >65 y (n = 1205)	1.49 (5.38)	0.04 (0.04)	0.02 (0.01)	93.6 (45.1)	7.5 (9.3)
Race/ethnicity^b^					
White non-Hispanic (n = 1983)	1.88 (5.92)	0.04 (0.04)	0.01 (0.01)	91.3 (41.9)	7.7 (9.1)
Black non-Hispanic (n = 67)	1.11 (2.19)	0.04 (0.04)	0.01 (0.01)	96.9 (51.9)	3.9 (8.6)
Hispanic (n = 44)	1.36 (5.53)	0.02 (0.02)	0.007 (0.005)	63.2 (22.7)	4.6 (7.4)
Other (n = 11)^c^	2.54 (4.58)	0.02 (0.01)	0.07 (0.003)	38.6 (25.7)	3.8 (4.6)
Mixed (n = 103)^d^	3.11 (4.72)	0.04 (0.03)	0.01 (0.01)	76.2 (51.2)	6.4 (6.9)
High school completion					
Low (<85%) (n = 945)	1.23 (4.22)	0.03 (0.04)	0.01 (0.01)	104.0 (51.1)	7.7 (10.4)
High (≥85%) (n = 1263)	2.37 (6.58)	0.04 (0.04)	0.02 (0.01)	79.5 (31.5)	7.3 (7.7)
Unemployment					
Low (<5%) (n = 140)	0.98 (2.41)	0.04 (0.04)	0.02 (0.02)	65.4 (34.3)	3.7 (5.9)
High (≥5%) (n = 2068)	1.97 (5.91)	0.04 (0.04)	0.01 (0.01)	91.7 (42.8)	7.7 (9.1)
Poverty					
Low (<10%) (n = 370)	2.65 (7.32)	0.04 (0.04)	0.01 (0.01)	65.1 (27.6)	6.2 (6.9)
High (≥10%) (n = 1838)	1.77 (5.41)	0.04 (0.04)	0.01 (0.01)	95.0 (43.6)	7.7 (9.3)
Median household income					
Low (<$60 000) (n = 1922)	1.70 (5.49)	0.04 (0.04)	0.01 (0.01)	93.9 (43.9)	7.5 (9.3)
High (≥$60 000) (n = 286)	3.20 (7.18)	0.04 (0.03)	0.01 (0.01)	63.7 (20.3)	7.2 (6.5)
Income inequality^e^					
Low (Gini coefficient <0.4) (n = 242)	2.04 (5.99)	0.04 (0.04)	0.01 (0.01)	92.4 (43.3)	6.1 (7.1)
High (Gini coefficient ≥0.4) (n = 1966)	0.92 (3.50)	0.03 (0.03)	0.01 (0.01)	69.81 (31.7)	7.6 (9.2)
Metropolitan area					
Metropolitan (n = 1033)	2.81 (6.63)	0.04 (0.04)	0.01 (0.01)	82.5 (34.6)	8.0 (8.6)
Nonmetropolitan (n = 1175)	0.94 (4.50)	0.03 (0.04)	0.01 (0.01)	96.6 (47.9)	6.9 (9.3)
Census region					
South (n = 1042)	1.86 (5.48)	0.04 (0.04)	0.01 (0.01)	104.2 (48.4)	8.4 (10.4)
Midwest (n = 679)	1.55 (6.31)	0.04 (0.04)	0.02 (0.01)	79.7 (35.1)	5.5 (7.0)
West (n = 280)	1.66 (4.43)	0.03 (0.03)	0.01 (0.01)	80.3 (30.6)	6.9 (7.0)
Northeast (n = 207)	3.54 (6.74)	0.04 (0.04)	0.01 (0.01)	66.1 (19.1)	9.8 (8.2)

^a^ N = 2208; counties that did not receive any pharmaceutical industry marketing are not included.

^b^ Classified according to race/ethnicity exceeding 50% of the county composition.

^c^ Other counties are those with 50% of individuals or more identified as non-Hispanic Asian, American Indian or Alaskan Native, or Pacific Islander.

^d^ Mixed counties are those that did not meet a 50% threshold for white, black, Hispanic, or other (non-Hispanic Asian, American Indian or Alaskan Native, or Pacific Islander) race/ethnicity.

^e^ Gini index of income inequality ranges from 0, representing perfect income equality (ie, all incomes within a county are the same), to 1, representing perfect inequality (ie, 1 individual within a county holds all the county’s income, and all others in the same county have no income).^20,21,22,23^

Table 2 shows the association between opioid marketing and subsequent deaths from prescription opioid overdoses across counties.^20,21,22,23^ After adjustment for county-level covariates, overdose mortality was significantly associated with all 3 measures of opioid marketing. The greatest effect size was observed for the number of payments per capita, and the smallest effect size was observed for the marketing value in dollars per capita; mortality from opioid overdoses increased with each 1-SD increase in marketing value in dollars per capita (aRR, 1.09; 95% CI, 1.05-1.12), number of payments to physicians per capita (aRR, 1.18; 95% CI, 1.14-1.21), and number of physicians receiving marketing per capita (aRR, 1.12; 95% CI, 1.08-1.16). Overdose mortality was also significantly associated with all county-level covariates except for being aged 65 years or older.

**Table 2.** Association of Pharmaceutical Company Opioid Marketing With Prescription Opioid Overdose Deaths Across All US Counties^a^

Characteristic	aRR (95% CI)^b^		
	Model A	Model B	Model C
Marketing value, $ per 1000 population per year	1.09 (1.05-1.12)	NA	NA
No. of payments, per 1000 population per year	NA	1.18 (1.14-1.21)	NA
No. of physicians receiving payments, per 1000 population per year	NA	NA	1.12 (1.08-1.16)
Age group, %			
18-34 y	1.05 (1.03-1.07)	1.04 (1.02-1.06)	1.05 (1.03-1.06)
35-64 y	1.10 (1.07-1.12)	1.09 (1.07-1.12)	1.09 (1.07-1.12)
≥65 y	1.01 (0.99-1.02)	1.01 (0.99-1.02)	1.01 (1.00-1.03)
Male, %	0.93 (0.91-0.95)	0.94 (0.92-0.96)	0.94 (0.92-0.96)
White, %	1.01 (1.01-1.02)	1.01 (1.01-1.02)	1.01 (1.01-1.02)
High school or lower education, %	1.00 (1.00-1.01)	1.00 (1.00-1.01)	1.00 (1.00-1.01)
Unemployment, %	1.03 (1.01-1.04)	1.03 (1.02-1.05)	1.03 (1.01-1.04)
Poverty, %	1.03 (1.01-1.04)	1.03 (1.01-1.04)	1.03 (1.01-1.04)
Median household income ($1000)	1.00 (1.00-1.01)	1.00 (1.00-1.01)	1.00 (1.00-1.01)
Gini index^c^	1.01 (1.00-1.02)	1.00 (1.00-1.02)	1.01 (1.00-1.02)
Metropolitan area	1.21 (1.11-1.31)	1.13 (1.04-1.22)	1.20 (1.10-1.30)

Abbreviations: aRR, adjusted relative risk; NA, not applicable.

^a^ N = 9398 county-years for each analysis.

^b^ Model A includes marketing value (in dollars) as the independent variable, model B includes number of payments as the independent variable, and model C includes number of physicians receiving payments as the independent variable. Each model also includes all other covariates listed in the table.

^c^ Gini index of income inequality ranges from 0, representing perfect income equality (ie, all incomes within a county are the same), to 1, representing perfect inequality (ie, 1 individual within a county holds all the county’s income, and all others in the same county have no income).^20,21,22,23^

Table 3 displays the association between opioid marketing and subsequent opioid prescribing rates across counties.^20,21,22,23^ After adjustment for county-level covariates, opioid prescribing rates were significantly associated with all 3 measures of opioid marketing. As for mortality rates from prescription opioid overdoses, the greatest effect size in association with opioid prescribing rates was observed for the number of payments per capita, and the smallest effect size was observed for the marketing value in dollars per capita. Opioid prescribing rates increased with each 1-SD increase in marketing value in dollars per capita (aRR, 1.82; 95% CI, 1.00-2.65), number of payments per capita (aRR, 11.08; 95% CI, 9.31-12.86), and number of physicians receiving marketing per capita (aRR, 13.59; 95% CI, 11.48-15.71). Overdose mortality was also significantly associated with all county-level covariates except for the age ranges of 18 to 34 years and 35 to 64 years (for 2 of 3 marketing measures), educational level, and poverty.

**Table 3.** Association of Pharmaceutical Company Opioid Marketing With Opioid Prescribing Rates (per 100 Population) Across US Counties^a^

Characteristic	aRR (95% CI)^b^		
	Model A	Model B	Model C
Marketing value, $ per 1000 population per year	1.82 (1.00 to 2.65)	NA	NA
No. of payments, per 1000 population per year	NA	11.08 (9.31 to 12.86)	NA
No. of physicians receiving payments, per 1000 population per year	NA	NA	13.59 (11.48 to 15.71)
Age group, %			
18-34 y	1.10 (0.23 to 1.97)	0.70 (−0.13 to 1.53)	0.64 (−0.17 to 1.44)
35-64 y	1.82 (0.90 to 2.75)	1.58 (0.73 to 2.43)	1.52 (0.75 to 2.30)
≥65 y	−1.78 (−2.52 to −1.04)	−1.86 (−2.58 to −1.14)	−1.70 (−2.39 to −1.00)
Male, %	−4.70 (−5.84 to −3.56)	−4.04 (−5.05 to −3.03)	−3.78 (−4.68 to −2.89)
White, %	0.65 (0.54 to 0.76)	0.60 (0.49 to 0.71)	0.54 (0.43 to 0.65)
High school or lower education, %	−0.08 (−0.30 to 0.14)	0.04 (−0.17 to 0.25)	0.07 (−0.14 to 0.28)
Unemployment, %	2.73 (2.10 to 3.35)	2.56 (1.96 to 3.16)	2.40 (1.80 to 2.99)
Poverty, %	0.02 (−0.59 to 0.63)	0.17 (−0.41 to 0.75)	0.30 (−0.27 to 0.87)
Median household income ($1000)	−1.01 (−1.29 to −0.73)	−0.98 (−1.24 to −0.72)	−0.89 (−1.14 to −0.64)
Gini index^c^	1.39 (0.76 to 2.01)	0.90 (0.30 to 1.50)	0.89 (0.30 to 1.48)
Metropolitan area	−4.85 (−8.76 to −0.94)	−9.49 (−13.41 to −5.575)	−8.04 (−11.59 to −4.48)

Abbreviations: aRR, adjusted relative risk; NA, not applicable.

^a^ N = 8885 county-years for each analysis; opioid prescribing rates were missing for 513 county-years (5.8%).

^b^ Model A includes marketing value (in dollars) as the independent variable, model B includes number of payments as the independent variable, and model C includes number of physicians receiving payments as the independent variable. Each model also includes all other covariates listed in the table.

^c^ Gini index of income inequality ranges from 0, representing perfect income equality (ie, all incomes within a county are the same), to 1, representing perfect inequality (ie, 1 individual within a county holds all the county’s income, and all others in the same county have no income).^20,21,22,23^

Table 4 shows the results of the mediation analysis to determine whether opioid prescribing rates were a potential mediator of the association between opioid marketing and mortality rates from prescription opioid overdoses. The strongest mediating effects were observed for the number of physicians who received payments, and the smallest effect size was observed for the marketing value in dollars per capita. Prescribing rates mediated 3% of the association between opioid marketing and mortality from overdoses in terms of value in dollars per capita, 11% of the association between opioid marketing and mortality from overdoses in terms of number of payments per capita, and 26% of the association between opioid marketing and mortality from overdoses in terms of number of physicians receiving marketing per capita. Findings were consistent in sensitivity analyses in which multiple imputation was used to generate values for counties missing data on opioid prescribing (eTables 2-4 in the Supplement).

**Table 4.** Mediation Analysis of Opioid Prescribing Rate as an Intermediate in the Association Between Pharmaceutical Company Opioid Marketing and Mortality From Prescription Opioid Overdoses Across US Counties

Characteristic	Natural Direct Effect (95% CI)^a^	Natural Indirect Effect (95% CI)^b^	Total Effect (95% CI)	% Mediated
Marketing value, $ per 1000 population per year	1.43 (1.36-1.50)	1.01 (1.01-1.02)	1.44 (1.37-1.52)	3
No. of payments, per 1000 population per year	1.50 (1.44-1.56)	1.05 (1.04-1.06)	1.57 (1.51-1.64)	11
No. of physicians receiving payments, per 1000 population per year	1.22 (1.16-1.28)	1.07 (1.06-1.09)	1.31 (1.25-1.37)	26

^a^ Natural direct effect measures the expected increase in deaths from prescription opioid overdoses as opioid marketing increases while setting prescribing rates to the value they would have attained before opioid marketing increased.

^b^ Natural indirect effect measures the expected increase in deaths from prescription opioid overdoses when opioid marketing is held constant at its baseline level and prescribing rates change to whatever value they would have attained (for each county) with an increase in opioid marketing.

## Discussion

In this national study of the association between pharmaceutical company marketing of opioids to physicians and deaths from prescription opioid overdoses, we found that counties receiving such marketing subsequently experienced elevated mortality. In addition, opioid prescribing rates were strongly associated with the burden of opioid marketing across counties and partly mediated the association between marketing and deaths from opioid overdoses. These findings held for multiple different measures of opioid marketing across counties, including the total dollar amount of marketing received, the number of payments made per capita, and the number of physicians receiving any marketing per capita.

Our study adds to recent literature suggesting that pharmaceutical company marketing of specific products may be associated with increased prescribing of those medications.^9,10^ Recent data suggest that when physicians receive opioid marketing, they subsequently prescribe more opioids.^11^ We build on prior studies, however, by identifying an association of opioid marketing with deaths from opioid overdoses. Although there remains the possibility of reverse causality—that is, that counties with high opioid prescribing rates and already experiencing elevated mortality from overdoses are subsequently targeted by pharmaceutical company marketing—it is potentially concerning that physicians in such counties would receive further marketing for opioids.

The pharmaceutical industry invests tens of millions of dollars annually in direct-to-physician marketing of opioids,^12^ and it is improbable that companies would provide payments to physicians if such marketing did not either increase prescribing rates or maintain high levels of opioid prescribing. Although we were able to examine data only from 2013 onwards, substantial attention has been focused on the extent to which pharmaceutical companies’ marketing practices may have contributed to the early stages of the opioid overdose crisis, during which time prescription opioids were in oversupply and were the most common cause of deaths from opioid overdoses.^30^ Today, opioid-related overdoses in the United States increasingly involve heroin, illicitly manufactured fentanyl, and numerous other substances such as alcohol, benzodiazepines, and cocaine.^2,31^ Nonetheless, the United States continues to vastly exceed the rest of the developed world in opioid prescribing, and many individuals with opioid use disorder are first introduced to opioids through a prescription.^3,4^ Our findings suggest that direct-to-physician opioid marketing may counter current national efforts to reduce the number of opioids prescribed^7^ and that policymakers might consider limits on these activities as part of a robust, evidence-based response to the opioid overdose epidemic in the United States.

There have been calls for limits on drug company marketing to physicians,^32^ which may reduce unnecessary prescribing.^33^ New Jersey, for example, recently passed a regulation to limit total payments to physicians at $10 000 annually.^34^ However, we found that the number of promotional payments made and the number of physicians receiving payments may have more of an association with county-level opioid prescribing rates and, in turn, with mortality from opioid overdoses than the dollar value of marketing. Because most marketing interactions with physicians involve meals that typically have a low monetary value,^35^ a high dollar cap would affect only a minority of prescribers who exceed this amount.^11^ As evidence mounts that industry-sponsored meals contribute to increased prescribing,^9,10,11^ data suggest that the greatest influence of pharmaceutical companies may be subtle and widespread, manifested through payments of low monetary value occurring on a very large scale.

### Limitations

This study has several limitations. First, as outlined earlier, our findings demonstrate associations between opioid marketing and subsequent prescribing and mortality from overdoses; we cannot exclude reverse causation. Nonetheless, we build on prior cross-sectional marketing studies by lagging marketing from prescribing and deaths from overdoses.^9,10^ Second, although we examine the outcome of deaths related to prescription opioid overdoses, many of these deaths also include the involvement of heroin, fentanyl, or other highly potent opioids, as well as other substances such as alcohol, benzodiazepines, or cocaine.^2,31^ Our analyses do not examine the larger outcome of all deaths related to opioid overdoses (ie, including heroin, fentanyl, and other highly potent illicit opioid analogs), nor do they explicitly examine associations between marketing and polysubstance use–related deaths, which should be areas for future research. In addition, the data sources used do not allow for the differentiation between opioids that are prescribed and those that are illicitly acquired. Third, because systematic data on marketing have only recently become available, we were unable to examine long-term trends. The potential effects of marketing on opioid prescribing and deaths from overdoses are likely to be the result of repeated industry interactions occurring over many years; the potential cumulative effect of marketing should be the subject of future studies. Fourth, with the measures of opioid prescribing used, we are unable to distinguish appropriate opioid prescribing from potentially inappropriate prescribing. Similarly, the Open Payments database does not include further information on the nature of industry-physician interactions; it is possible that some industry payments to physicians resulted in improved knowledge around safe prescribing practices.^36^

## Conclusions

Prescription opioids continue to contribute to more than 17 000 deaths from overdoses annually in the United States.^2^ Amid a worsening opioid crisis, our results suggest that industry marketing to physicians may run counter to current efforts to curb excessive opioid prescribing. Policymakers should continue to consider limiting the extent to which pharmaceutical companies may contribute to inappropriate opioid prescribing while balancing the need for access to opioids for patients who need them. Pharmaceutical companies might also consider, as one manufacturer recently did,^37^ voluntarily ceasing marketing of opioid products directly to physicians. Professional medical organizations and licensing boards should continue to support education to help physicians prescribe opioids appropriately and consider the use of nonpharmacologic and nonopioid analgesics.^25^ Reducing the number of opioids prescribed is only 1 facet of a much-needed, multipronged public health effort to reduce opioid-related harm^38^ and is unlikely to reduce the current high mortality rate from overdoses that are attributable to heroin and illicitly manufactured fentanyl.^39^ Nonetheless, further clarifying the limits of drug companies’ influence on physician prescribing should be a critical component of preventing the current crisis from worsening.

## Notes

Supplement. **eTable 1.** Characteristics of Counties According to Receipt of any Opioid Marketing in 2015 **eTable 2.** Association of Pharmaceutical Company Opioid Marketing With Prescription Opioid Overdose Deaths Across US Counties, After Using Multiple Imputation for Counties With Missing Opioid Prescribing Rates **eTable 3.** Association of Pharmaceutical Company Opioid Marketing With Opioid Prescribing Rates (Per 100 Population) Across US Counties, After Using Multiple Imputation for Counties With Missing Opioid Prescribing Rates **eTable 4.** Mediation Analysis of Opioid Prescribing Rate as an Intermediate in the Association Between Pharmaceutical Company Opioid Marketing and Prescription Opioid Overdose Mortality Across US Counties After Using Multiple Imputation for Counties With Missing Opioid Prescribing Rates
