# Errors in Results, Table 3, and eTables 2 and 3

**DOI:** 10.1001/jamanetworkopen.2019.1625

**Published:** 2019-03-22

In the Original Investigation titled “Association of Pharmaceutical Industry Marketing of Opioid Products With Mortality From Opioid-Related Overdoses,”^1^ published January 18, 2019, the adjusted relative risk (aRR) and 95% CI for the number of payments per capita were inadvertently switched with those for the number of physicians receiving marketing per capita in the second to last paragraph of the Results section and in the 2 columns representing model B and model C in Table 3. The effect sizes for the 2 main independent variables in the 2 columns in Table 3 are comparable (number of payments per 1000 population per year: aRR, 11.08; 95% CI, 9.31-12.86; number of physicians receiving payments per 1000 population per year: aRR, 13.59; 95% CI, 11.48-15.71). Thus, the study’s conclusions remain unchanged. Also, from “Age group” on down to “Metropolitan area,” in the 2 columns representing model B and model C in Table 3, the aRRs and 95% CIs were inadvertently switched. Similarly, eTable 3 had columns that were inadvertently switched but with comparable effect sizes, again leaving the study’s conclusions unchanged. Finally, the title of eTable 2 has been corrected to better reflect that death counts rather than mortality rates were used as a dependent variable in statistical modeling. This article has been corrected.^1^

## References

[1] Hadland SE, Rivera-Aguirre A, Marshall BDL, Cerdá M. Association of pharmaceutical industry marketing of opioid products with mortality from opioid-related overdoses. JAMA Netw Open. 2019;2(1):e186007. doi:10.1001/jamanetworkopen.2018.6007 30657529 PMC6484875

