# Supplementary material for: Association of Pharmaceutical Industry Marketing of Opioid Products With Mortality From Opioid-Related Overdoses
Source: JAMA Netw Open. 2019 Jan 18;2(1):e186007. doi: 10.1001/jamanetworkopen.2018.6007 (PMC6484875; doi:10.1001/jamanetworkopen.2018.6007)
Supplement: Supplement. — eTable 1. Characteristics of Counties According to Receipt of any Opioid Marketing in 2015 eTable 2. Association of Pharmaceutical Company Opioid Marketing With Prescription Opioid Overdose Deaths Across US Counties, After Using Multiple Imputation for Counties With Missing Opioid Prescribing Rates eTable 3. Association of Pharmaceutical Company Opioid Marketing With Opioid Prescribing Rates (Per 100 Population) Across US Counties, After Using Multiple Imputation for Counties With Missing Opioid Prescribing Rates eTable 4. Mediation Analysis of Opioid Prescribing Rate as an Intermediate in the Association Between Pharmaceutical Company Opioid Marketing and Prescription Opioid Overdose Mortality Across US Counties After Using Multiple Imputation for Counties With Missing Opioid Prescribing Rates [file jamanetwopen-e186007-s001.pdf]

Supplementary Online Content

Hadland SE, Rivera-Aguirre A, Marshall BDL, Cerdá M. Association of pharmaceutical industry marketing of opioid products with mortality from opioid-related overdoses. *JAMA Netw Open.* 2019;2(1):e186007. doi:10.1001/jamanetworkopen.2018.6007

eTable 1. Characteristics of Counties According to Receipt of any Opioid Marketing in 2015
eTable 2. Association of Pharmaceutical Company Opioid Marketing With Prescription Opioid Overdose Deaths Across US Counties, After Using Multiple Imputation for Counties With Missing Opioid Prescribing Rates
eTable 3. Association of Pharmaceutical Company Opioid Marketing With Opioid Prescribing Rates (Per 100 Population) Across US Counties, After Using Multiple Imputation for Counties With Missing Opioid Prescribing Rates
eTable 4. Mediation Analysis of Opioid Prescribing Rate as an Intermediate in the Association Between Pharmaceutical Company Opioid Marketing and Prescription Opioid Overdose Mortality Across US Counties After Using Multiple Imputation for Counties With Missing Opioid Prescribing Rates

This supplementary material has been provided by the authors to give readers additional information about their work.

© 2018 Hadland SE et al. *JAMA Network Open.*

eTable 1. Characteristics of Counties According to Receipt of any Opioid Marketing in 2015[a]

**Characteristic**	**Did Not Receive Marketing, *N* (%)**	**Received Any Marketing, *N* (%)**
Race/ethnicity[b]		
White non-Hispanic (*N* = 2,796)	1,031 (36.9%)	1,765 (63.1%)
Black non-Hispanic (*N* = 98)	44 (44.9%)	54 (55.1%)
Hispanic (*N* = 89)	53 (59.6%)	36 (40.5%)
Other[c] (*N* = 35)	27 (77.1%)	8 (22.9%)
Mixed[d] (*N* = 124)	27 (21.8%)	97 (78.2%)
Age		
<15% over age 65 yrs (*N* = 1,247)	314 (25.2%)	933 (74.8%)
≥15% over age 65 yrs (*N* = 1,895)	868 (45.8%)	1,027 (54.2%)
High school completion		
Low <85%, (*N* = 1,402)	589 (42%)	813 (58%)
High, ≥85% (*N* = 1,740)	593 (34%)	1,147 (66%)
Unemployment		
Low, <5% (*N* = 432)	335 (77.6%)	97 (22.5%)
High, ≥5% (*N* = 2,710)	847 (31.3%)	1,863 (68.7%)
Poverty		
Low, <10% (*N* = 578)	256 (44.3%)	322 (55.7%)
High, ≥10% (*N* = 2,564)	926 (36.1%)	1,638 (63.9%)
Median household income		
Low, <$60,000 (*N* = 2,797)	1,099 (39.3%)	1,698 (60.7%)
High, ≥$60,000 (*N* = 345)	83 (24.1%)	75.9 (74.3%)
Income inequality[e]		
Low, Gini coefficient <0.4 (*N* = 395)	184 (46.6%)	211 (53.4%)
High, Gini coefficient ≥0.4 (*N* = 2,747)	998 (36.3%)	1,749 63.7%
Metropolitan area		
Metropolitan (*N* = 1,166)	176 (15.1%)	990 (84.9%)
Non-metropolitan (*N* = 1,976)	1,006 (50.9%)	970 (49.1%)
Census region		
South (*N* = 1,422)	494 (34.7%)	928 (65.3%)
Midwest (*N* = 1,055)	465 (44.1%)	590 (55.9%)

© 2018 Hadland SE et al. *JAMA Network Open.*

West (*N* = 448)	204<br>(45.5%)	244<br>(54.5%)
Northeast (*N* = 217)	19<br>(8.8%)	198<br>(91.2%)

a. *N* = 3,142
b. Classified according to race/ethnicity exceeding 50% of the county composition
c. ‘Other’ counties are those with ≥50% of individuals identified as non-Hispanic Asian, American Indian or Alaskan Native, or Pacific Islander
d. ‘Mixed’ counties are those that did not meet a 50% threshold for white, black, Hispanic or other (non-Hispanic Asian, American Indian or Alaskan Native, or Pacific Islander) race/ethnicity
e. Gini index of income inequality ranges from zero, representing perfect income equality (i.e., all incomes within a county are the same), to 1, representing perfect inequality (i.e., one individual within a county holds all the county’s income, and all others in the same county have zero income)[17]

© 2018 Hadland SE et al. *JAMA Network Open.*

eTable 2. Association of Pharmaceutical Company Opioid Marketing With Prescription Opioid Overdose Deaths Across US Counties, After Using Multiple Imputation for Counties With Missing Opioid Prescribing Rates[a]

Characteristic	aRR	95% CI	aRR	95% CI	aRR	95% CI
Marketing value ($ per 1,000 pop. per month)	1.09	(1.05, 1.12)				
Number of payments (per 1,000 pop. per month)			1.17	(1.14, 1.21)		
Number of physicians receiving payments (per 1,000 pop. per month)					1.11	(1.07, 1.16)
Age 18-34 %	1.05	(1.02, 1.08)	1.05	(1.02, 1.08)	1.05	(1.02, 1.08)
Age 35-64%	1.10	(1.07, 1.13)	1.10	(1.07, 1.13)	1.10	(1.07, 1.13)
Age 65+%	1.01	(0.99, 1.04)	1.01	(0.99, 1.03)	1.02	(0.99, 1.04)
Male %	0.93	(0.91, 0.96)	0.94	(0.92, 0.96)	0.94	(0.92, 0.96)
White %	1.01	(1.01, 1.02)	1.01	(1.01, 1.01)	1.01	(1.01, 1.02)
HS or less %	1.00	(1.00, 1.01)	1.00	(1.00, 1.01)	1.00	(1.00, 1.01)
Unemployment %	1.02	(1.00, 1.04)	1.02	(1.01, 1.04)	1.02	(1.00, 1.04)
Poverty %	1.03	(1.01, 1.04)	1.03	(1.01, 1.04)	1.03	(1.01, 1.04)
Median household income (1,000 dollars)	1.00	(1.00, 1.01)	1.00	(1.00, 1.01)	1.00	(1.00, 1.01)
Gini index[b]	1.01	(0.99, 1.02)	1.00	(0.98, 1.02)	1.01	(0.99, 1.02)
Metropolitan area	1.21	(1.12, 1.32)	1.13	(1.04, 1.24)	1.20	(1.10, 1.31)

a. *N* = 9,420 county-years

b. Gini index of income inequality ranges from zero, representing perfect income equality (i.e., all incomes within a county are the same), to 1, representing perfect inequality (i.e., one individual within a county holds all the county's income, and all others in the same county have zero income)[17]

© 2018 Hadland SE et al. *JAMA Network Open.*

eTable 3. Association of Pharmaceutical Company Opioid Marketing With Opioid Prescribing Rates (Per 100 Population) Across US Counties, After Using Multiple Imputation for Counties With Missing Opioid Prescribing Rates[a]

Characteristic	aRR	95% CI	aRR	95% CI	aRR	95% CI
Marketing value ($ per 1,000 pop. per month)	1.80	(0.96, 2.64)				
Number of payments (per 1,000 pop. per month)			11.01	(9.22, 12.79)		
Number of physicians receiving payments (per 1,000 pop. per month)					13.46	(11.34, 15.59)
Age 18-34 %	1.69	(0.79, 2.59)	1.28	(0.40, 2.16)	1.19	(0.32, 2.06)
Age 35-64%	2.53	(1.60, 3.45)	2.30	(1.42, 3.18)	2.25	(1.41, 3.08)
Age 65+%	-1.31	(-2.06, -0.56)	-1.36	(-2.10, -0.62)	-1.22	(-1.94, -0.50)
Male %	-4.62	(-5.70, -3.54)	-3.99	(-4.97, -3.01)	-3.74	(-4.63, -2.84)
White %	0.54	(0.42, 0.65)	0.49	(0.37, 0.60)	0.43	(0.32, 0.54)
HS or less %	0.00	(-0.23, 0.23)	0.10	(-0.12, 0.32)	0.12	(-0.10, 0.34)
Unemployment %	2.39	(1.77, 3.00)	2.20	(1.60, 2.80)	2.04	(1.45, 2.64)
Poverty %	0.09	(-0.50, 0.67)	0.21	(-0.35, 0.77)	0.33	(-0.22, 0.89)
Median household income (1,000 dollars)	-0.96	(-1.23, -0.68)	-0.94	(-1.20, -0.68)	-0.86	(-1.12, -0.61)
Gini index[b]	1.03	(0.41, 1.65)	0.58	(-0.02, 1.19)	0.58	(-0.02, 1.18)
Metropolitan area	-5.34	(-9.21, -1.48)	-9.93	(-13.82, -6.04)	-8.63	(-12.20, -5.07)

a. *N* = 9,420 county-years

b. Gini index of income inequality ranges from zero, representing perfect income equality (i.e., all incomes within a county are the same), to 1, representing perfect inequality (i.e., one individual within a county holds all the county’s income, and all others in the same county have zero income)[17]

© 2018 Hadland SE et al. *JAMA Network Open.*

eTable 4. Mediation Analysis of Opioid Prescribing Rate as an Intermediate in the Association Between Pharmaceutical Company Opioid Marketing and Prescription Opioid Overdose Mortality Across US Counties After Using Multiple Imputation for Counties With Missing Opioid Prescribing Rates

Characteristic	Natural direct effect[a]	95% CI	Natural indirect effect[b]	95% CI	Total Effect	95% CI	% Mediated
Marketing value ($ per 1,000 pop. per month)	1.46	(1.35, 1.57)	1.01	(1.01, 1.02)	1.48	(1.36, 1.59)	3%
Number of payments (per 1,000 pop. per month)	1.55	(1.48, 1.63)	1.05	(1.04, 1.07)	1.64	(1.56, 1.71)	10%
Number of physicians receiving payments (per 1,000 pop. per month)	1.28	(1.22, 1.35)	1.07	(1.06, 1.09)	1.37	(1.31, 1.44)	22%

a. Natural direct effect measures the expected increase in prescription opioid overdose deaths as opioid marketing increases, while setting prescribing rates to the value they would have attained before opioid marketing increased.

b. Natural indirect effect measures the expected increase in prescription opioid overdose deaths when opioid marketing is held constant at its baseline level, and prescribing rates change to whatever value they would have attained (for each county) with an increase in opioid marketing.

© 2018 Hadland SE et al. *JAMA Network Open*.
